# Macroporous Hydroxyapatite-Based Bone Scaffolds Loaded with CAPE Derivatives: A Strategy to Reduce Oxidative Stress and Biofilm Formation

**DOI:** 10.3390/ma18225074

**Published:** 2025-11-07

**Authors:** Paulina Kazimierczak, Marwa Balaha, Krzysztof Palka, Joanna Wessely-Szponder, Michal Wojcik, Viviana di Giacomo, Barbara De Filippis, Agata Przekora

**Affiliations:** 1 Department of Tissue Engineering and Regenerative Medicine, Medical University of Lublin, 20-093 Lublin, Poland; michal.wojcik@up.lublin.pl (M.W.); agata.przekora@umlub.pl (A.P.); 2 Department of Pharmacy, University “G. d’Annunzio” of Chieti-Pescara, 66100 Chieti, Italy; marwa.balaha@unich.it (M.B.); viviana.digiacomo@unich.it (V.d.G.); barbara.defilippis@unich.it (B.D.F.); 3 Department of Pharmaceutical Chemistry, Kafrelsheikh University, Kafr El Sheikh 33516, Egypt; 4 Department of Materials Engineering, Lublin University of Technology, 20-618 Lublin, Poland; k.palka@pollub.pl; 5 Sub-Department of Pathophysiology, Department of Preclinical Veterinary Sciences, University of Life Sciences in Lublin, 20-950 Lublin, Poland; joanna.wessely@up.lublin.pl; 6 UdA-TechLab, Research Center, University “G. d’Annunzio” of Chieti-Pescara, 66100 Chieti, Italy

**Keywords:** biomaterial, osteoblast, cytotoxicity, biocompatibility, bioactivity, porosity

## Abstract

Caffeic acid phenethyl ester (CAPE), a polyphenol from propolis, is well recognized for its anti-inflammatory, antioxidant, antimicrobial, and osteogenic properties. This study aimed to develop macroporous bone scaffolds composed of a chitosan/agarose matrix reinforced with nanohydroxyapatite and enriched with stable CAPE derivatives to enhance their biomedical potential for applications in bone tissue engineering and regenerative medicine. A comprehensive evaluation of microstructural and biological properties of the produced scaffolds was conducted. The fabricated scaffolds exhibited high porosity (49–60%) with interconnected pores and compressive strength (1.2–1.8 MPa), closely resembling cancellous bone and indicating suitability for bone regeneration. They were biocompatible, promoted osteoblast adhesion, proliferation, and differentiation, and supported apatite deposition on their surfaces, demonstrating strong bioactivity and potential for implant osseointegration. Importantly, the scaffolds did not trigger excessive production of reactive oxygen or nitrogen species, suggesting a low risk of inflammatory responses. Additionally, CAPE-enriched scaffolds inhibited biofilm formation by *Staphylococcus aureus* and *Staphylococcus epidermidis*, reducing the risk of implant-associated infections. In summary, these CAPE-modified scaffolds integrate optimal microstructural and biological features, such as reducing oxidative stress and inhibiting biofilm formation, and thus offer a promising strategy for enhancing bone repair and regeneration in clinical applications.

## 1. Introduction

Reconstruction of bone defects still remains a major challenge, particularly with comorbidities such as osteoporosis, diabetes and chronic stress, which can impair the bone’s regenerative capacity [1,2]. Bone tissue-engineered constructs represent alternative approach to conventional treatments for bone defects, such as autografts and allografts. These constructs typically utilize scaffolds with a three-dimensional porous architecture and pore sizes that support cell migration, vascularization, and bone ingrowth, while also providing sufficient strength and stiffness to stabilize the defect site. An effective approach involves enriching biomaterials with biological agents or signals to enhance progenitor cell adhesion, proliferation, differentiation, as well as vascular infiltration, thereby improving their osteoconductive and osteoinductive properties [2,3,4]. Bioactivation of bone tissue-engineered constructs can be achieved in two ways: (i) by incorporating bioactive molecules, including proteins, peptides, ions, nanoparticles, pharmacological agents, phytochemicals, platelet-rich plasma, and fibrin; or (ii) by seeding with cells [1,3,5].

Caffeic acid phenethyl ester (CAPE) is a polyphenolic active component found in propolis, a honeybee-derived resin [6]. It has been comprehensively studied and shown to exhibit an extensive range of biological properties, including anti-inflammatory (often mediated through inhibition of NF-κB signaling) and immunomodulatory properties [7], and antiviral, antibacterial [8,9], and antifungal activities [6]. It was also demonstrated in preclinical (cell culture and animal) models that CAPE has antiproliferative/anticancer effects with mechanisms such as histone deacetylase inhibition and modulation of oncogenic pathways. Moreover, it shows antioxidant activity, functioning to scavenge free radicals and reduce oxidative stress [7,10,11]. Additionally, numerous animal studies have reported that CAPE enhances bone regeneration and repair, underscoring its potential as a remedial agent in bone healing [12]. Indeed, evidence indicates that CAPE supports bone preservation and restoration through actions on both osteoblasts and osteoclasts. In osteoblasts, CAPE counteracts oxidative stress-induced suppression of differentiation markers such as bone alkaline phosphatase (bALP), type I collagen, colony-forming unit osteoprogenitors, and RUNX2 nuclear phosphorylation [12]. Regarding osteoclasts, CAPE exerts a dual regulatory role: at low concentrations it inhibits osteoclastogenesis by blocking RANKL-mediated NF-κB and NFAT activation, while at higher doses it promotes apoptosis and disrupts the microtubule organization in osteoclast-like cells [12].

**Figure 1:**
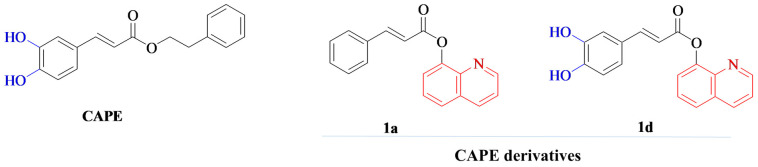
Chemical structures of caffeic acid phenethyl ester (CAPE) and its synthesized derivatives (1a and 1d) used in this study. The synthesis and characterization of these derivatives were described in our previous work [6].

Despite CAPE’s promising therapeutic potential, its clinical application is hindered by its poor stability as it is highly susceptible to hydrolysis both in vivo and in vitro, which impairs its efficacy and bioavailability [13,14]. Its degradation is further accelerated by elevated temperatures and alkaline pH, while storage at lower temperatures and mildly acidic conditions can slow this process [13]. To overcome these limitations, various chemical modifications have been explored. Strategies such as introducing fluorine into the catechol ring [13] or replacing the ester bond with an amide bond have yielded analogs with improved thermal and enzymatic stability, enhanced bioavailability, and retained cytoprotective activity [15]. In our previous research, novel hybrid derivatives were synthesized by combining the cinnamic or caffeic acid moiety with quinoline or isoquinoline via an ester or amide linker. These compounds exhibited enhanced stability and wound-healing capacity, highlighting their potential for applications in regenerative medicine [6]. Among these, compounds marked as 1a and 1d (designations consistent with our previous work on their synthesis [6]; Figure 1) demonstrated the most effective wound-healing activity as well as superior chemical and thermal stability [6]. Therefore, they were selected for further investigation of their bone regenerative potential.

The goal of this study was to synthesize highly macroporous bone scaffolds comprising a chitosan/agarose matrix, strengthened with nanohydroxyapatite and enriched with synthesized, highly stable CAPE derivatives (1a and 1d—the same designations were used in our previous research on their synthesis [6]) to enhance their biomedical potential for the regeneration of bone tissue. The biomaterials were fabricated according to the procedure developed by our team and disclosed in Polish Patent no. 235822. This patented method, which combines a gas foaming method with a lyophilization process, allows the production of highly macroporous scaffolds for cancellous bone repair [16]. Our previous studies confirmed that the developed macroporous chitosan/agarose/nanohydroxyapatite biomaterial was biocompatible, osteoconductive, and osteoinductive. Moreover, this biomaterial exhibited high liquid absorption capacity, reaching equilibrium rapidly, and was capable of biodegradation in enzymatic degradation solution (containing lysozyme and collagenase), non-enzymatic solutions at physiological pH 7.4 (PBS and Tris-HCl), and in an acidic environment (citric acid, pH 3) [16,17]. These properties make it a promising candidate for bone regeneration and reconstruction. Notably, based on the available literature, no prior reports exist describing bone scaffolds containing a bioactive agent such as CAPE. Therefore, the results of this study may provide novel insights into the use of CAPE derivatives to boost the biological performance of bone tissue-engineered constructs and increase their clinical utility.

A comparative evaluation of the cellular response to nanohydroxyapatite-based bone scaffolds enriched with the two CAPE derivatives (1a and 1d) was performed using human osteoblasts through cytotoxicity, cell proliferation, as well as osteogenic differentiation assays. In addition, the generation of reactive oxygen species (ROS) and reactive nitrogen species (RNS) by human immune cells, as well as biofilm formation, was assessed. The scaffolds were also evaluated for their microstructure, mechanical strength, and ability to form apatite on the surface.

## 2. Materials and Methods

### 2.1. Materials

Chitosan, agarose, nanohydroxyapatite, sodium bicarbonate, DMEM/Ham’s F12 medium without phenol red, streptomycin, penicillin, G418, ascorbic acid, β-glycerophosphate, dexamethasone, Live/Dead Double Staining Kit, Total Lactate Dehydrogenase (LDH) Assay, paraformaldehyde, Triton X-100, DAPI were bought from Sigma-Aldrich Chemicals (St. Louis, MO, USA). Fetal bovine serum was purchased from Pan-Biotech GmbH (Aidenbach, Bavaria, Germany). Simulated body fluid (SBF) was purchased from Biochemazone (Edmonton, AB, Canada). AlexaFluor635-conjugated phalloidin was bought from Invitrogen (Carlsbad, CA, USA). Mueller-Hinton agar was purchased from Biomaxima (Lublin, Poland). BCA Protein Assay Kit was purchased from ThermoFisher Scientific (Waltham, MA, USA). Acetic acid solution was bought from Avantor Performance Materials (Gliwice, Poland). A Viability/Cytotoxicity Assay Kit for Bacteria Live & Dead Cells was bought from Biotium (Fremont, CA, USA).

### 2.2. Fabrication of Bone Scaffolds

The preparation of biomaterials was carried out based on Polish Patent no. 235822 and prior reports [16,17]. Briefly, 2% (*w*/*v*) chitosan (59.8 kDa, 85% degree of deacetylation) and 5% (*w*/*v*) agarose (6.5 kDa) were suspended in a 2% (*v*/*v*) acetic acid solution containing the CAPE derivatives that were synthesized as described earlier [6], i.e., 1a at a concentration of 0.45 µg/mL and 1d at a concentration of 0.49 µg/mL (sample marked Mat_1a and Mat_1d, respectively). A control scaffold without bioactive compounds (Mat_control) was also prepared. Subsequently, 40% (*w*/*v*) hydroxyapatite (HA) and 2% (*w*/*v*) sodium bicarbonate were added and mixed. The resulting pastes were put in the molds and heated at 95 °C for 15 min, cooled, frozen at −80 °C, and finally lyophilized (Alpha 1-4 LSCbasic, Martin Christ Gefriertrocknungsanlagen GmbH, Osterode am Harz, Germany). Before conducting any experiments, scaffolds were sterilized through immersion in 70% (*v*/*v*) ethanol, followed by rinsing with deionized water and air-drying.

### 2.3. Microstructure Characterization

The microstructure of the fabricated bone implants was examined using a stereoscopic microscope (SZ61TR, Olympus, Tokyo, Japan) and a scanning electron microscope (SEM; JEOL JCM-6000Plus, Tokyo, Japan). Porosity was assessed by micro-computed tomography (micro-CT; Xradia 510 Versa, Carl Zeiss X-ray Microscopy, Inc., Dublin, CA, USA) at a resolution of 5.77 µm. Total porosity was quantified using Dragonfly ORS (Comet Technologies Canada Inc., Montréal, QC, Canada). The three-dimensional surface topography and surface roughness of the samples were analyzed using a confocal laser scanning optical profilometer (Olympus LEXT OLS5100, Tokyo, Japan). For each biomaterial, nine measurements were taken from different surface regions (1288.3 µm × 1288.3 µm) at a magnification of 210×, and the areal surface roughness (S_a_) was subsequently calculated.

### 2.4. Determination of Mechanical Properties

Compressive strength and Young’s modulus were measured using an Autograph AG-X Plus testing machine (Shimadzu Corp., Kyoto, Japan) with a crosshead speed of 1 mm/min and a load cell accuracy of 0.1 N. Compressive stress was recorded at 30% strain.

### 2.5. Liquid Absorption Ability

The capacity of the biomaterials to absorb liquid was assessed by monitoring the change in their weight over time after immersion in PBS. The extent of fluid uptake was represented as the percentage increase in weight (*W_i_*), determined using the following equation: *W_i_* = (*W_t_* − *W*_0_)/*W*_0_ × 100. In this formula, *W*_0_ denotes the initial dry weight of the sample, and *W_t_* corresponds to the sample’s weight at a given time t [18].

### 2.6. Bioactivity Assessment

The apatite-forming capacity of the biomaterials was evaluated following the ISO 23317:2025 protocol [19]. Samples were immersed in SBF and maintained at 37 °C for 28 days. Following incubation, the formation of apatite deposits on the biomaterial surfaces was examined using scanning electron microscopy (SEM; Zeiss ULTRA plus, New York, NY, USA) equipped with an Octane Pro EDS detector (EDAX) (Carl Zeiss Microscopy, LLC, White Plains, New York, NY, USA). The presence of apatite precipitates was verified by energy-dispersive spectroscopy (EDS) through calculation of the Ca/P atomic ratio.

### 2.7. Evaluation of Biological Properties In Vitro

#### 2.7.1. Cells

A normal human fetal osteoblast cell line (hFOB 1.19; ATCC-LGC Standards, Teddington, UK) was employed to evaluate cytotoxicity, cell proliferation, and osteogenic differentiation on the surfaces of the fabricated bone scaffolds. hFOB 1.19 cells were maintained in a 1:1 DMEM/Ham’s F12 medium supplemented with 10% (*v*/*v*) fetal bovine serum, 1% (*v*/*v*) streptomycin/penicillin solution, and 300 μg/mL G418. Osteoblasts were cultured at 34 °C in a humidified atmosphere containing 5% CO_2_ in air. For osteogenic differentiation, cells were cultured in osteogenic medium consisting of complete culture medium containing 0.05 mg/mL ascorbic acid, 0.01 M β-glycerophosphate, and 10^−8^ M dexamethasone, and incubated at 37 °C in a humidified atmosphere containing 5% CO_2_.

ROS and RNS production induced by the biomaterials was assessed using immune cells (neutrophils, monocytes) isolated from human peripheral blood (Bioethics Committee approval no. KE-0254/187/10/2022). Isolation of neutrophils and monocytes was performed following previously described protocols [20].

#### 2.7.2. Cytotoxicity Assessment

Cytotoxicity estimation was carried out using the MTT assay according to ISO 10993-5 [21], and biomaterial extracts were prepared according to ISO 10993-12 guidelines [22]. Briefly, extracts were made by immersing 100 mg of each biomaterial in 1 mL of culture medium and incubating at 37 °C for 24 h. Culture medium incubated in polystyrene wells without biomaterials (designated as control) served as the negative control for cytotoxicity. A total of 2 × 10^4^ hFOB 1.19 cells per well were seeded into 96-well plates and cultured at 34 °C for 24 h. The cells were then treated with 100 μL of the respective biomaterial extracts and incubated for an additional 24 h. Cell viability was estimated using the MTT colorimetric assay. Data were presented as a percentage of the optical density (OD) measured for the control. Additionally, the concentration of CAPE derivatives in the obtained extracts was evaluated by UV–Vis absorbance measurements at 328 nm using standard curves prepared for compounds 1a and 1d.

Furthermore, a direct contact cytotoxicity test was carried out. hFOB 1.19 cells were directly seeded at 6 × 10^4^ cells per sample onto biomaterials that had been placed in 48-well plates and prewetted with medium. After 72 h of incubation, cells were stained using the Live/Dead Double Staining Kit according to the manufacturer’s instructions and observed with a confocal laser scanning microscope (CLSM, Olympus Fluoview with FV1000, Olympus, Tokyo, Japan).

#### 2.7.3. Cell Proliferation Assessment

hFOB 1.19 cells (6 × 10^4^ per sample) were directly seeded onto biomaterials placed in 48-well plates and wetted with medium. Cell numbers were quantified after 1, 2, and 3 days by measuring total LDH activity following lysis, according to the manufacturer’s protocol. The cell numbers were calculated based on a calibration curve generated from known concentrations of hFOB 1.19 cells. Additionally, after 3 days of culture on the biomaterial surfaces, osteoblast morphology was analyzed through fluorescent staining and CLSM. Prior to imaging, cells were fixed with paraformaldehyde and permeabilized with Triton X-100. Actin filaments were then stained with AlexaFluor635-conjugated phalloidin, while nuclei were stained with DAPI.

#### 2.7.4. Osteogenic Differentiation

Biomaterials were placed in the wells of a 48-well plate and wetted with medium. The hFOB 1.19 were seeded directly onto the biomaterial samples at a density of 8 × 10^4^ cells per sample and cultured at 37 °C in osteogenic medium for a period of 21 days. To evaluate the osteogenic differentiation process in vitro, the production of the three osteogenic differentiation markers—type 1 collagen, bone sialoprotein 2, and osteocalcin—was determined in cell lysates. Quantification of these markers was performed using commercially available ELISA kits (EIAab, Wuhan, China). Cell lysates were prepared according to a two-step procedure: two consecutive freeze–thaw cycles to induce membrane disruption, followed by sonication at 30% amplitude. The concentrations of osteogenic markers obtained from ELISA assays were normalized to the total cellular protein content, which was quantified using the BCA Protein Assay Kit. Final results were expressed as nanograms (ng) or picograms (pg) of osteogenic marker per milligram (mg) of total cellular protein.

In addition, after 21 days of osteoblast culture on the biomaterial surfaces, the cells were visualized using SEM (JEOL JCM-6000Plus, Tokyo, Japan). Before SEM observation, the samples were fixed as described in Section 2.7.3 and dehydrated in increasing ethanol concentrations—35%, 50%, 75%, 95%, and 99.8%—for 10 min per concentration, followed by two final immersions in 99.8%. The dried biomaterials were sputter-coated with a thin gold layer (7 nm) under high vacuum using a vacuum sputter (Vac Coat Sputter Coater DSR1, London, UK) and examined by SEM. SEM micrographs were acquired under high vacuum at an accelerating voltage of 5 kV.

#### 2.7.5. ROS/RNS Generation by Immune Cells

Neutrophils and monocytes (1 × 10^6^ cells) were seeded onto the biomaterials, and cells grown in 24-well plates without scaffolds served as the control group. After 24 h of immune cell culture, ROS/RNS generation was assessed. Superoxide (O_2_^−^) and nitrite (NO_2_^−^) production were evaluated using previously described colorimetric methods [20], employing nitroblue tetrazolium solution and the Griess reaction, respectively.

#### 2.7.6. Evaluation of Biofilm Formation

Reference bacterial strains *Staphylococcus aureus* ATCC 25923 and *Staphylococcus epidermidis* ATCC 12228 were employed for the biofilm formation assay. Biomaterial samples were immersed in 20-fold diluted Mueller–Hinton broth and inoculated with bacterial suspensions of *S. aureus* and *S. epidermidis* at a concentration of approximately 5 × 10^5^ CFU/mL. The samples were subsequently incubated at 36 °C for 24 h. Following incubation, the scaffolds were gently rinsed with PBS to remove non-adherent cells and stained using the Viability/Cytotoxicity Assay Kit for Bacteria Live & Dead Cells, according to the manufacturer’s instructions. The green-fluorescent dye labels all bacteria, regardless of membrane integrity, whereas the red-fluorescent dye specifically marks non-viable bacteria with damaged membranes.

### 2.8. Statistical Analysis

Data were presented as mean ± standard deviation (SD) from a minimum of three independent experiments (*n* = 3). Statistical analyses were conducted using one-way analysis of variance (ANOVA) followed by Tukey’s test (*p* < 0.05) using GraphPad Prism software (version 8.0.0; GraphPad Software Inc., San Diego, CA, USA) to compare each experimental group with the others.

## 3. Results and Discussion

### 3.1. Characterization of Microstructure, Mechanical Properties, Liquid Absorption Ability, and Bioactivity

**Figure 2:**
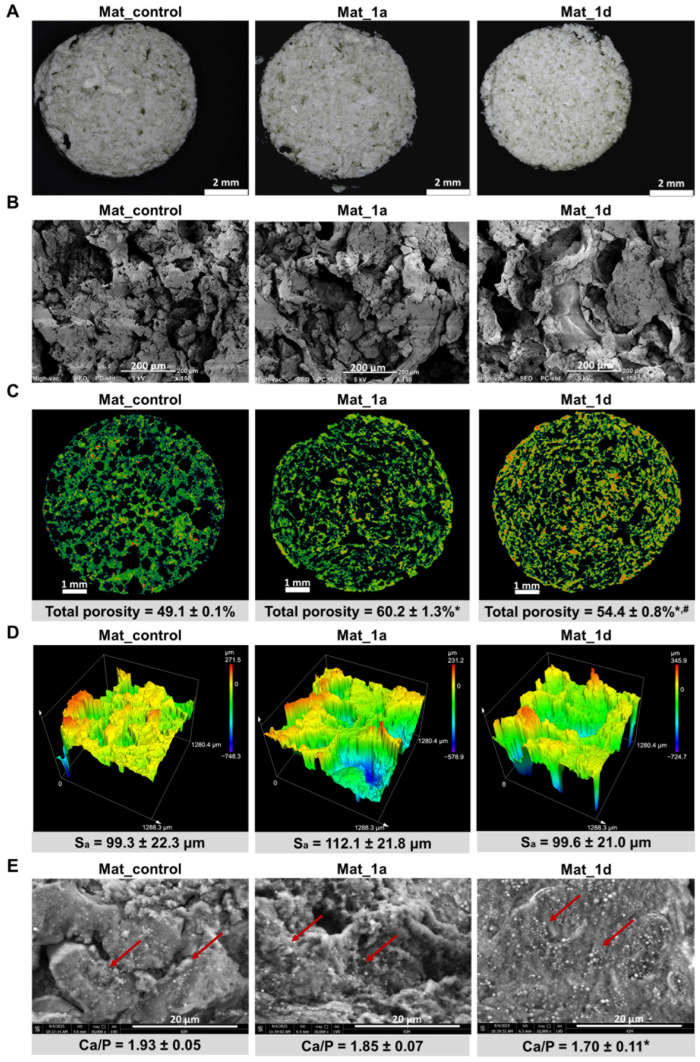
(**A**–**D**) Microstructural characterization of the fabricated biomaterials using (**A**) stereoscopic microscope (scale bar: 2 mm), (**B**) SEM (150× magnification, scale bar: 200 μm), (**C**) micro-CT (cross-sectional images and porosity analysis; scale bar: 1 mm), and (**D**) confocal laser scanning optical profilometer (surface topography and areal surface roughness (S_a_ in µm) were measured); (**E**) bioactivity assessment via SEM-EDS analysis of the surface after 28 days of immersion in simulated body fluid (10,000× magnification, scale bar: 20 μm; red arrows indicate apatite crystals formed on the surface of the tested biomaterials). Statistical analysis: * statistically significant results compared to Mat_control, ^#^ statistically significant results compared to Mat_1a; one-way analysis ANOVA followed by Tukey’s test.

The microstructure of bone scaffolds is a critical determinant of their performance in bone tissue engineering, as it governs cellular responses, tissue ingrowth, and mechanical stability. This refers to the micrometer-scale internal architecture, which is key in regulating cell adhesion, proliferation, differentiation, and nutrient transport [4]. Figure 2 presents visualizations of the surface topography and microstructure of the fabricated biomaterials. Images obtained with a stereoscopic microscope (Figure 2A) confirmed that the biomaterials were porous and exhibited a rough surface, features known to enhance cell attachment and osteogenic differentiation [23]. In turn, SEM analysis revealed a macroporous microstructure (Figure 2B), which is essential for facilitating vascularization and bone tissue ingrowth [4]. Furthermore, micro-CT visualization demonstrated the presence of a well-interconnected pore network (Figure 2C), a characteristic that ensures efficient nutrient and oxygen diffusion throughout the scaffold and supports long-term tissue viability [24]. Micro-CT analysis of porosity revealed that Mat_control, Mat_1a, and Mat_1d exhibited total porosities of 49.1%, 60.2%, and 54.4%, respectively. Such porosity levels are within the range considered to be favorable for bone regeneration, enhancing cell infiltration, vascularization, and nutrient diffusion, whereas excessively high porosity may decrease mechanical stability [2,24]. Moreover, the Mat_1a material was characterized by the highest porosity among the tested samples. It can be assumed that the incorporation of 1a derivative slightly decreased the viscosity of the polymers and hydroxyapatite mixture during production, promoting the formation of greater porosity as a result of gas-foaming process. Nevertheless, to verify these assumptions, further chemical analyses are required.

The surface topography of the bone scaffolds was evaluated using a confocal laser scanning optical profilometer, revealing S_a_ values between 99.3 and 112.1 μm (Figure 2D). Based on the Albrektsson and Wennerberg classification [25], all materials were categorized as rough (S_a_ > 2 μm). Surface roughness critically influences cellular behavior, for example, osteoblasts typically favor rougher surfaces, while epithelial cells prefer smoother ones [26].

The liquid absorption capacity of the biomaterials was evaluated in PBS. All tested materials reached absorption equilibrium within 5 min of immersion (*W_i_* = 157.5 ± 25.7%, 165.6 ± 23.2%, and 154.2 ± 35.7% for Mat_control, Mat_1a, and Mat_1d, respectively). The sorption behavior was comparable among all samples and no statistically significant differences were observed (one-way ANOVA followed by Tukey’s post hoc test, *p* > 0.05). After 24 h, the *W_i_* values remained in the range of 163.0–167.6%, confirming that absorption equilibrium was maintained. Pre-soaking biomaterials in liquids such as blood plasma or saline is a common preoperative step; however, this process should be kept relatively short to minimize the risk of contamination and to prevent excessive swelling of the implant at the implantation site. The fabricated biomaterials exhibited compressive strengths of 1.5 ± 0.5 MPa (Mat_control), 1.8 ± 0.6 MPa (Mat_1a), and 1.2 ± 0.4 MPa (Mat_1d). In turn, the Young’s modulus values were low: 16.3 ± 8.3 MPa (Mat_control), 19.9 ± 4.3 MPa (Mat_1a), and 15.9 ± 5.8 MPa (Mat_1d), indicating high elasticity. No statistically significant differences among the samples were observed in the mechanical behavior tests (one-way ANOVA followed by Tukey’s post hoc test, *p* < 0.05). Taking into account that human cancellous bone exhibits a Young’s modulus of 0.1–0.5 GPa and a compressive strength of 1.9–12 MPa [27], it can be suggested that compressive strength values of the scaffolds were close to the lower range of cancellous bone, but remained significantly more flexible. This mechanical profile suggests suitability for non-load-bearing implantation area or application with additional mechanical support using internal fixation devices such as wires, plates, or screws.

Bioactivity, defined as the ability of a biomaterial to form apatite crystals on its surface, is a critical property that enables strong bonding with surrounding bone tissue and facilitates osseointegration [28,29]. The in vitro apatite-forming capacity of biomaterials can be evaluated by immersion in SBF, which mimics the ionic composition of blood plasma [29,30]. After 28 days of incubation in SBF, all fabricated biomaterials induced apatite formation on their surfaces (Figure 2D). The crystals displayed globular to hemispherical morphologies, highlighted by red arrows in the SEM images. EDS analysis confirmed that observed deposits are calcium phosphates, with Ca/P atomic ratios of 1.93 ± 0.05 (Mat_control), 1.85 ± 0.07 (Mat_1a), and 1.70 ± 0.11 (Mat_1d). The measured Ca/P ratios (1.93, 1.85, and 1.70) fall within the range typically reported for bone-like apatite layers, with values slightly higher than the stoichiometric ratio of natural hydroxyapatite (1.67) likely reflecting the presence of calcium-rich phases (e.g., CaO, CaCO_3_, or poorly crystallized apatite) [30]. The result for Mat_1d (1.70) was particularly close to the theoretical value, indicating that this material may promote the formation of apatite with chemistry very similar to natural bone mineral. Overall, these findings demonstrate that all scaffolds are bioactive, capable of inducing bone-like apatite nucleation and growth, which is essential for integration with host bone. The slight differences among groups might also point to variations in surface chemistry, porosity, or ion release influencing the apatite precipitation process.

### 3.2. Evaluation of Biological Properties In Vitro

#### 3.2.1. Cytotoxicity and Cell Proliferation Assessment

Beyond fulfilling the necessary microstructural and physicochemical requirements, bone implant materials must also demonstrate essential biological properties, notably biocompatibility and osteoconductivity, in order to enhance tissue regeneration at the implantation site. Biocompatibility is defined as the capacity of a biomaterial to elicit appropriate cellular responses without causing toxic, genotoxic, or immunogenic effects. In contrast, osteoconductivity refers to a biomaterial’s ability to support cell adhesion, proliferation, and the production of bone extracellular matrix (ECM) [31,32,33].

**Figure 3:**
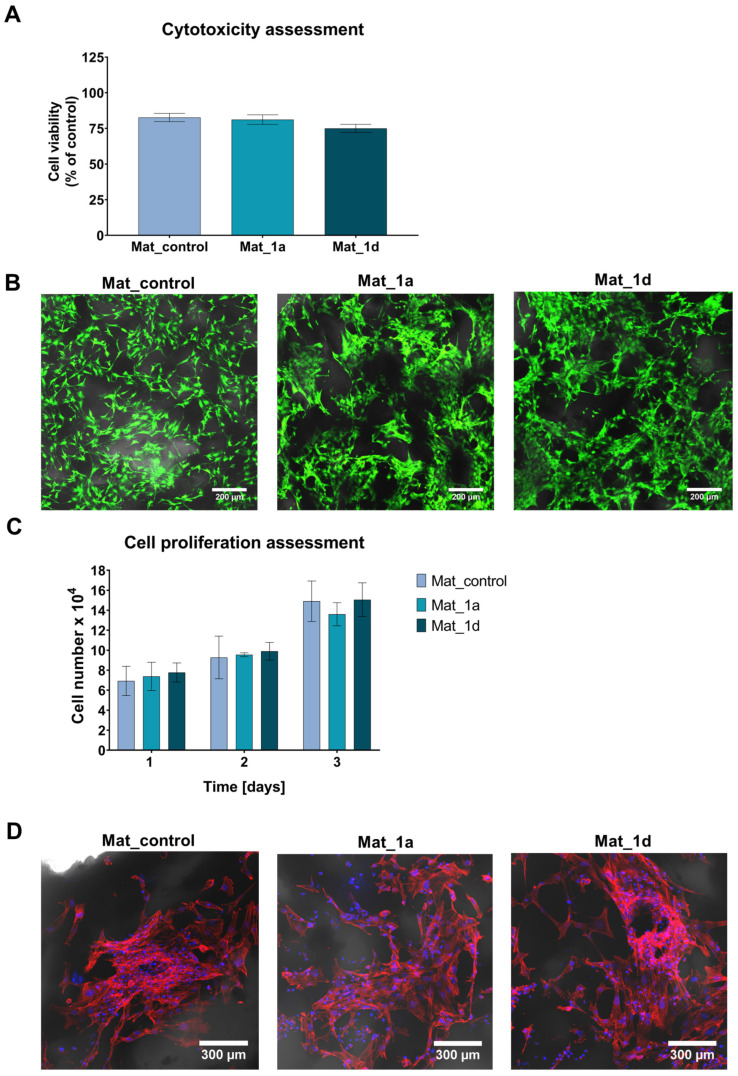
Evaluation of cellular response to fabricated biomaterials: (**A**) Cytotoxicity assessment of the fabricated biomaterials against hFOB 1.19 cells according to ISO 10993-5 using the MTT assay; (**B**) Cytotoxicity assessment in direct contact with biomaterials, followed by confocal laser scanning microscopy visualization of cells after live/dead staining (green fluorescence—viable cells; red fluorescence—dead cells; Nomarski contrast was applied to visualize biomaterials; magnification: 100×; scale bar = 200 μm); (**C**) Cell proliferation evaluation on the surface of the fabricated biomaterials using the Total LDH assay; (**D**) Confocal laser scanning microscope images showing fluorescent staining of the cell cytoskeleton after 3-day cell culture on the surface of biomaterials (red fluorescence—actin filament, blue fluorescence—nuclei; Nomarski contrast was applied to visualize biomaterials; magnification: 200×; scale bar = 300 µm).

In this study, cytotoxicity of the fabricated biomaterials was assessed by the MTT assay and live/dead staining using human fetal osteoblast cell line (hFOB 1.19), a well-established model due to its similarity to primary osteoblasts. The MTT assay demonstrated that all tested biomaterials were non-toxic againts hFOB 1.19 cells (Figure 3A). Cell viability after exposure to 24 h extracts of the biomaterials exceeded 75%. Notably, according to ISO 10993-5, cell viability above 70% following exposure to 100% biomaterial extract is considered indicative of a non-toxic effect. The slight reduction in cell viability observed is most likely attributable to alterations in the ionic composition of the culture medium, as evidenced by the lack of significant differences between Mat_control and biomaterials enriched with CAPE derivatives. Calcium phosphate bioceramics represent a fundamental component of numerous bone scaffolds and commercially available biomaterials. It is well established that surface-reactive biomaterials can alter the concentrations of essential ions in the surrounding microenvironment, thereby influencing cellular metabolism and viability [34]. Furthermore, the concentration of CAPE derivatives in the 24 h extracts was quantified. In the biomaterial extracts, it was determined that 37.2% (0.11 µg/mL) of 1a was released from Mat_1a and 45.5% (0.15 µg/mL) of 1d was released from Mat_1d.

Figure 3B presents live/dead staining of osteoblasts cultured on the surfaces of the fabricated biomaterials. The CLSM images demonstrated good adhesion of numerous viable cells (green fluorescence) to the scaffold surfaces, confirming their non-cytotoxic nature. Moreover, the number of osteoblasts on the surfaces of the fabricated bone scaffolds was quantified at 1, 2, and 3 days post-seeding using the Total LDH assay. As shown in Figure 3C, the fabricated biomaterials supported progressive cell proliferation over time; however, the presence of CAPE derivatives had no significant effect on this process. Additionally, fluorescent staining of the cell cytoskeleton after 3 days of culture revealed a high density of cells with well-developed cytoskeletal structures, further demonstrating the osteoconductive properties of the scaffolds (Figure 3D).

#### 3.2.2. Osteogenic Differentiation Assessment

To ensure effective bone formation in vivo, it is desirable for newly developed bone scaffolds to promote the osteogenic differentiation of osteoprogenitor cells or mesenchymal stem cells. Osteogenic differentiation occurs in three primary stages, each associated with characteristic molecular markers: (1) cell proliferation, marked by RUNX2, type I collagen, and fibronectin, with minimal bALP activity; (2) ECM synthesis and maturation, characterized by elevated bALP activity, type I collagen, and Osterix expression; and (3) ECM mineralization, indicated by the presence of osteocalcin, osteopontin, osteonectin, bone sialoproteins, and moderate bALP activity [31,35,36].

**Figure 4:**
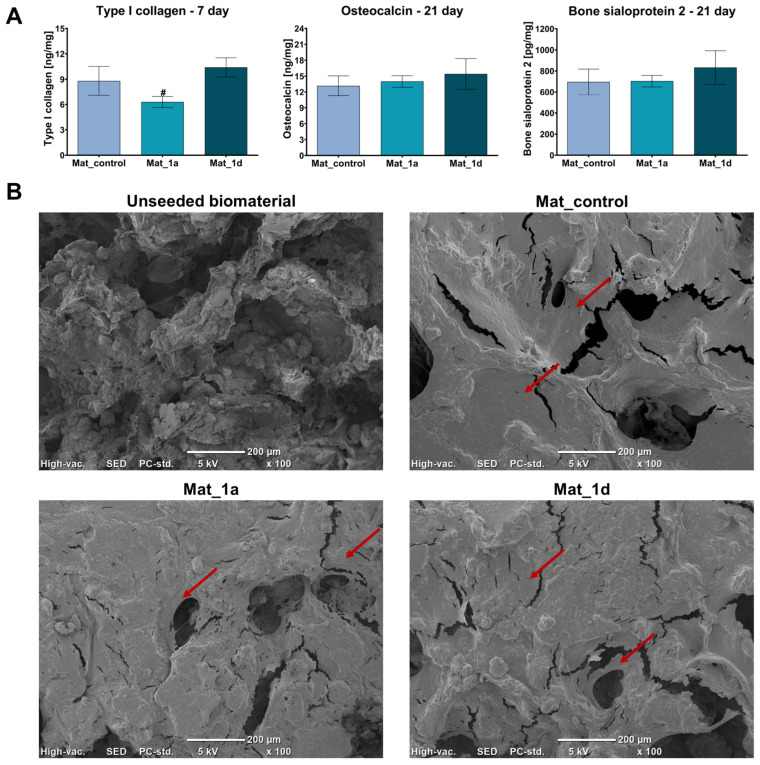
Evaluation of osteogenic differentiation of hFOB 1.19 cells cultured on the surface of biomaterials: (**A**) Levels of osteogenic markers determined by ELISA (^#^ statistically significant compared to Mat_1d; one-way analysis ANOVA followed by Tukey’s test); (**B**) SEM images of cells on the surface of biomaterials after 21 days of culture in osteogenic medium (unseeded biomaterial is a representative sample of all biomaterials without osteoblast seeding; red arrows indicate cell sheets on the surface of the biomaterials).

In this study, osteogenic differentiation of hFOB 1.19 cells cultured on the surfaces of the biomaterials was assessed by measuring osteogenic marker levels using ELISA (Figure 4A). Quantitative analysis of type I collagen synthesis after 7 days of culture revealed that Mat_1a significantly reduced collagen production, whereas Mat_1d slightly increased collagen synthesis compared to Mat_control, though this increase was not statistically significant. After 21 days of culture, the levels of osteocalcin and bone sialoprotein 2 were similar in cells cultured on the control biomaterial (Mat_control) and on biomaterials enriched with CAPE derivatives (Mat_1a and Mat_1d). Additionally, on day 21 of osteogenic differentiation, cells cultured on the surfaces of the fabricated biomaterials were visualized using SEM (Figure 4B). The SEM images revealed extensive sheets of well-spread cells, indicating both biocompatibility and osteoconductivity of the biomaterials. After the long-term culture, the scaffold surfaces were densely populated with cells, suggesting strong support for proliferation and new bone formation. Furthermore, cells wrapping around pores and extending along the surface topography indicate effective cell–biomaterial interactions and adaptation to the micro- and nanoscale features of the scaffolds.

According to the available literature, CAPE has been extensively studied in cell culture and animal models of bone remodeling. Its beneficial effects on new bone formation and bone healing have been comprehensively reported and summarized in the review by Ekeuku S. et al. [12]. Despite the promising results on biocompatibility and osteoconductivity, there was no striking evidence that compounds 1a and 1d significantly enhanced osteogenic differentiation. However, in another study from our group (data unpublished yet), 1a and 1d compounds—administered directly to a 2D culture of dental pulp stem cells—promoted osteogenic differentiation in an in vitro model of dental pulp stem cells by regulating alkaline phosphate activity, BMP2, and osterix gene expression. In the present study, the two compounds were incorporated into the microstructure of the biomaterial, which most likely limited their interactions with the surrounding cells and with key signaling pathways involved in osteogenesis (e.g., BMP/Smad, Wnt/β-catenin). Another possible reason for this discrepancy may be related to the insufficient release of 1a and 1d agents from the biomaterial to stimulate bone formation processes.

#### 3.2.3. Evaluation of ROS/RNS Generation by Human Immune Cells

Bone scaffolds for regenerative medicine applications must fulfill multiple biological functions to ensure successful integration and long-term performance. Ideally, they should support osteoblast adhesion, proliferation, and differentiation, thereby promoting new bone formation within the defect site. At the same time, they must not trigger adverse host responses, particularly chronic inflammation, which can disturb tissue regeneration and scaffold stability [31,37,38]. During the inflammatory phase following implantation, activated immune cells such as monocytes, macrophages, and neutrophils release excessive amounts of reactive oxygen species (ROS) and reactive nitrogen species (RNS). While these molecules play essential roles in host defense and wound healing at physiological levels, their overproduction can lead to oxidative stress, causing damage to cellular components, including lipids, proteins, and nucleic acids. Persistent oxidative stress in the local microenvironment may impair osteoblast function, hinder extracellular matrix mineralization, and ultimately delay or prevent proper bone regeneration. Moreover, prolonged oxidative stress may cause oxidative damage to the implant, further contributing to its instability and failure. As a result, the development of bone scaffolds has progressively focused on strategies aimed at modulating the immune response and reducing oxidative stress. Such strategies involve the integration of antioxidant compounds and/or therapeutic agents, the application of bioactive surface modifications, all intended to establish a local microenvironment that supports the transition from the initial inflammatory phase to effective tissue regeneration [31,38,39].

**Figure 5:**
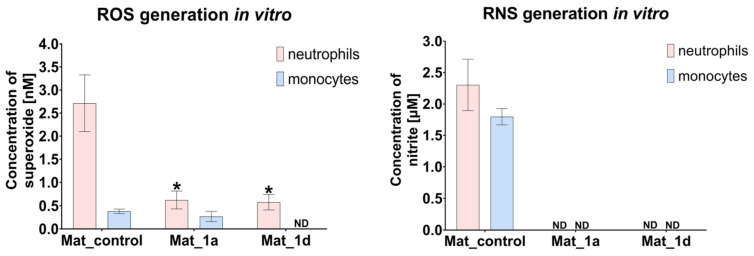
ROS/RNS production by human immune cells (* statistically significant results compared to Mat_control, one-way ANOVA followed by Tukey’s test).

The production of ROS and RNS by human immune cells, specifically neutrophils and monocytes, was assessed (Figure 5). Neutrophils cultured in the presence of Mat_1a and Mat_1d produced significantly lower levels of ROS compared to those cultured with Mat_control. ROS generation by monocytes was comparable between Mat_control and Mat_1a, whereas it was below the detection limit in cultures with Mat_1d. Similarly, RNS generation by neutrophils and monocytes in the presence of Mat_1a and Mat_1d was below the detection limit, suggesting a scavenging effect of the CAPE derivatives. Thus, it may be concluded that CAPE-enriched biomaterials present a very low risk of inducing chronic inflammation following implantation within a bone defect. Nevertheless, further investigations are necessary to comprehensively evaluate the inflammatory response to these scaffolds.

#### 3.2.4. Evaluation of Biofilm Formation

Implant-associated infections occur when bacteria colonize artificial medical devices, leading to inflammation, pain, swelling, and potential implant failure. Common causes include Staphylococcus species, especially *S. aureus* and *S. epidermidis* [35,36]. Bacterial colonization and subsequent biofilm formation remain major challenges in orthopedic implants. A bacterial biofilm is a structured community of microbial cells that irreversibly attaches to a surface and becomes embedded within a self-produced extracellular polymeric substance (EPS). The EPS matrix, primarily composed of polysaccharides, proteins, nucleic acids, and lipids, provides structural integrity to the biofilm and serves as a protective barrier against host immune defenses and antimicrobial agents. Preventing biofilm formation on orthopedic implants has therefore become a key focus in biomaterial research [40,41,42].

**Figure 6:**
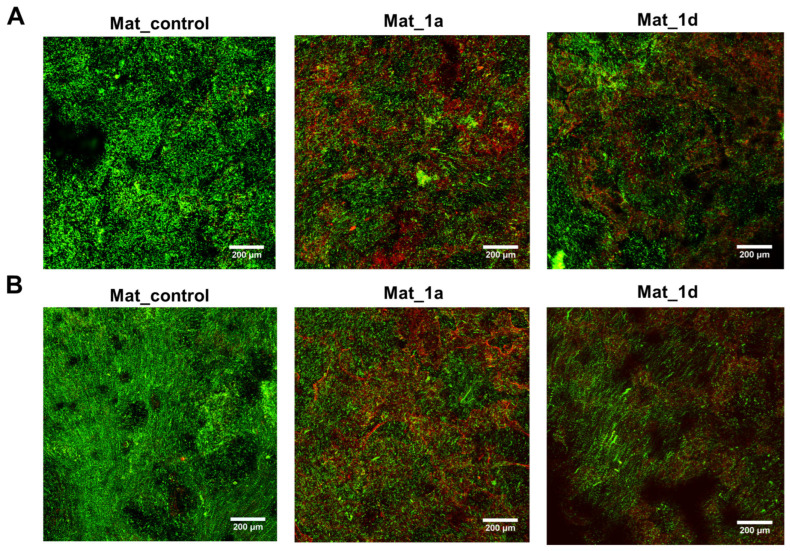
Confocal laser scanning microscopy images showing fluorescent staining of biofilm formed by (**A**) *Staphylococcus aureus* and (**B**) *Staphylococcus epidermidis* on biomaterials after 24 h (green fluorescence—viable and dead bacteria with intact cell membranes; red fluorescence—dead bacteria with damaged cell membranes; magnification: 100×; scale bar: 200 μm).

Figure 6 shows CLSM images of fluorescently stained biofilms of *S. aureus* (Figure 6A) and *S. epidermidis* (Figure 6B) on the surface of the biomaterials after 24 h of incubation with bacteria. Green fluorescence indicates both viable and dead bacteria with intact cell membranes, whereas red fluorescence marks only dead bacteria with damaged membranes. Mat_1a and Mat_1d exhibited antibacterial activity against both *S. aureus* and *S. epidermidis*, as evidenced by strong red fluorescence and a predominance of red over green signal. In contrast, for Mat_control, predominantly green fluorescence was observed, with only minor red signals, indicating that most bacteria remained viable [6]. These findings are consistent with our previous study by Balaha et al. [6], in which compounds 1a and 1d demonstrated antibacterial activity against *S. aureus* (MIC = 15.6 and 62.5 mg/L, respectively) and *S. epidermidis* (MIC = 7.8 and 31.3 mg/L, respectively), but showed no activity against Gram-negative bacteria, like *Escherichia coli*, *Pseudomonas aeruginosa* (MIC > 1000 mg/L). Furthermore, compounds 1a and 1d were also shown to inhibit fungal growth, with MICs of 15.6 mg/L and 31.25 mg/L, respectively, against *Candida albicans* and *Candida parapsilosis*.

Based on the available literature, the antibacterial action of CAPE is attributed primarily to their ability to disrupt bacterial membranes and interfere with quorum-sensing pathways [43,44]. These mechanisms may explain the pronounced activity of CAPE derivatives against Gram-positive bacteria, which have a less complex cell envelope compared to Gram-negative microorganisms [45]. It can therefore be assumed that the outer membrane of Gram-negative bacteria likely limits CAPE permeability and may account for the lack of antibacterial effect observed against E. coli and related strains in our previous work [6].

## 4. Conclusions

Chitosan/agarose/nanohydroxyapatite scaffolds enriched with stable CAPE derivatives exhibited microstructural and mechanical properties resembling cancellous bone, confirming their suitability for bone regeneration. The scaffolds demonstrated excellent biocompatibility and osteoconductivity, supporting osteoblast adhesion, proliferation, and differentiation, while also promoting apatite deposition indicative of strong bioactivity and potential for osseointegration. Importantly, CAPE enrichment not only decreased production of reactive oxygen or nitrogen species, suggesting a low risk of inflammatory responses, but also effectively inhibited bacterial biofilm formation. The integration of CAPE into macroporous scaffolds thus yields a multifunctional biomaterial that combines osteoconductive, bioactive, antioxidant, and antimicrobial properties, representing a promising platform for enhanced bone repair and regeneration in clinical applications. It can be concluded that the Mat_1a scaffold demonstrated the most balanced microstructural and biological performance among the studied biomaterials, making it the most promising candidate for bone grafting applications.

## 5. Patents

The method employed to fabricate the bone scaffolds is protected by Polish patent no. 235822.

## Data Availability

The original contributions presented in this study are included in the article. Further inquiries can be directed to the corresponding author.

## Abbreviations

The following abbreviations are used in this manuscript:

bALP	Bone Alkaline Phosphatase
CAPE	Caffeic Acid Phenethyl Ester
CLSM	Confocal Laser Scanning Microscope
ECM	Extracellular Matrix
EDS	Energy-Dispersive Spectroscopy
EPS	Extracellular Polymeric Substance
LDH	Lactate Dehydrogenase
RNS	Reactive Nitrogen Species
ROS	Reactive Oxygen Species
SBF	Simulated Body Fluid
